# Microbes from Brine Systems with Fluctuating Salinity Can Thrive under Simulated Martian Chemical Conditions

**DOI:** 10.3390/life12010012

**Published:** 2021-12-22

**Authors:** Matthew Kelbrick, James A. W. Oliver, Nisha K. Ramkissoon, Amy Dugdale, Ben P. Stephens, Ezgi Kucukkilic-Stephens, Susanne P. Schwenzer, André Antunes, Michael C. Macey

**Affiliations:** 1 Biology Department, Edge Hill University, Ormskirk L39 4QP, UK; 23187824@edgehill.ac.uk; 2 Department of Evolution, Ecology and Behaviour, Institute of Infection, Veterinary and Ecological Sciences, University of Liverpool, Liverpool L69 3GJ, UK; 3 AstrobiologyOU, School of Environment, Earth and Ecosystem Sciences, Faculty of Science, Technology, Engineering and Mathematics, The Open University, Milton Keynes MK7 6AA, UK; nisha.ramkissoon@open.ac.uk (N.K.R.); Ben.stephens@open.ac.uk (B.P.S.); ezgi.kucukkilic-stephens@open.ac.uk (E.K.-S.); susanne.schwenzer@open.ac.uk (S.P.S.); 4 AstrobiologyOU, School of Physical Sciences, Faculty of Science, Technology, Engineering and Mathematics, The Open University, Milton Keynes W23 F2H6, UK; amy.dugdale@open.ac.uk; 5 Biology Department, Maynooth University, Maynooth, W23 F2H6 Kildare, Ireland; 6 State Key Laboratory of Lunar and Planetary Sciences, Macau University of Science and Technology (MUST), Macau, China; aglantunes@must.edu.mo; 7 China National Space Administration (CNSA), Macau Center for Space Exploration and Science, Macau, China

**Keywords:** simulation, saline, martian, astrobiology, Mars

## Abstract

The waters that were present on early Mars may have been habitable. Characterising environments analogous to these waters and investigating the viability of their microbes under simulated martian chemical conditions is key to developing hypotheses on this habitability and potential biosignature formation. In this study, we examined the viability of microbes from the Anderton Brine Springs (United Kingdom) under simulated martian chemistries designed to simulate the chemical conditions of water that may have existed during the Hesperian. Associated changes in the fluid chemistries were also tested using inductively coupled plasma-optical emission spectroscopy (ICP-OES). The tested Hesperian fluid chemistries were shown to be habitable, supporting the growth of all of the Anderton Brine Spring isolates. However, inter and intra-generic variation was observed both in the ability of the isolates to tolerate more concentrated fluids and in their impact on the fluid chemistry. Therefore, whilst this study shows microbes from fluctuating brines can survive and grow in simulated martian water chemistry, further investigations are required to further define the potential habitability under past martian conditions.

## 1. Introduction

Water is essential to all known life. Although not the only prerequisite for habitability, it is the primary sign of potential past and present habitable environments outside of Earth. The majority of habitable martian environments are suggested to have existed early in Mars’s history, during the Noachian era (4.0–3.7 Gya) as surface water existed at that time [1,2,3,4]. However, some water sources are suggested to have persisted to the modern day (Amazonian era; 2.9 Gya to present), residing on the surface as evaporated streams or icecaps, and in the subsurface as groundwater in regions of high geothermal heat flux [3,5,6,7]. The historical presence of water on Mars is evidenced by geomorphological features, such as streambeds (e.g., [6]), and water-rock interactions documented by minerals that have undergone hydrous-alteration (e.g., [8,9,10])

The transition between the Noachian and the Hesperian eras (3.7–2.9 Gya) is characterised by decreased volcanism and atmospheric erosion, leading to a colder and dryer climate with increased evaporation, global cooling, and growth of the cryosphere [11,12]. Water activity was ongoing in spontaneous outflow channels, evidenced by floods [3,13,14]. The changes in water availability during this era, suggests that aqueous environments on the martian surface would have fluctuated in volume, chemistry, and water activity. Given the geological timescales over which these shifts would have occurred and the potential for accompanying evolutionary adaptations to the selection gradients, any inhabiting microbiota would require the genetic potential and phenotypic plasticity to withstand the spectrum of concentrations of salt experienced in the aqueous environments; whilst there are moderately halophilic microbes capable of inhabiting a broad range of salt concentrations, there are many extreme halophiles that are solely capable of survival in high salt concentrations [15,16], which would therefore be potentially excluded from the environments following decreases in concentration. Therefore, identifying microbes that can persist in environments analogous to the fluctuating conditions expected for the Noachian–Hesperian climate transition on Mars could be of use for furthering hypotheses with regard to habitability, viability, and biosignature formation on the Red Planet. Based on the salinity and fluctuating conditions of the Anderton Brine Springs, located within the Cheshire Salt District (UK), these were selected as potential appropriate analogue environments to characterise the impact of fluctuating concentrations of salts and ions on growth and survival. 

Anderton contains a series of interlocking brine springs that originate from subterranean Triassic evaporites exposed to groundwater that rises to the surface and gathers in local basins [17,18]. The Anderton Brine Springs undergo seasonal fluctuations in water level and salinity due to evaporation, drainage, rainfall, and their associated springs. These fluctuations impose a wide range of stresses on their inhabiting microbiome, selecting for a microbiome that is capable of survival in fluctuating environments. Here we show how novel microbial strains, retrieved from a series of brine springs with fluctuating salinity, survive and grow under varying strengths of simulated martian fluid chemistries. Furthermore, we investigate whether these isolates have an impact on the fluid chemistry that effects elements relevant to mineral formation, which could then be used to inform biosignature detection. 

This study contributes to a broad body of literature that has identified a range of halophilic and halotolerant microbes from highly saline environments that are identified as martian analogue [19,20,21,22] and that have investigated the survival and viability of microbes under a specific range of simulated martian conditions [23,24,25,26] environments. Our results support that environments with fluctuating salinity and their associated microbiomes represent suitable terrestrial ecosystems for the study of potential martian habitability. 

## 2. Materials and Methods

To identify the impact of varying martian fluid chemistry concentrations on microbial species, we isolated and identified microbes from the Anderton Brine Springs—a site chosen due to its fluctuating brine conditions. These isolates where grown in increasingly concentrated martian fluid chemistries to identify: (1) if fluid chemistry inhibits microbial growth; (2) if genus and strain level taxonomy determine an organism’s ability to grow under martian fluid chemistry; and (3) if microbial growth alters fluid chemistry in such a way that brine chemistry could be used to identify putative biosignatures that could subsequently form. The latter of which was tested using inductively coupled plasma-optical emission spectroscopy (ICP-OES). ICP-OES is an analytical technique that allows the detection and measurement of elements within a fluid. This technique was selected to measure dissolution of insoluble components of the supplied fluid chemistry, indicative of microbial metabolisms, and any potential decreases in specific elements within the fluid as a consequence of microbial metabolisms (e.g., the formation of precipitates removing specific elements from the fluid).

### 2.1. Sample Collection and Characterisation

**Figure 1:**
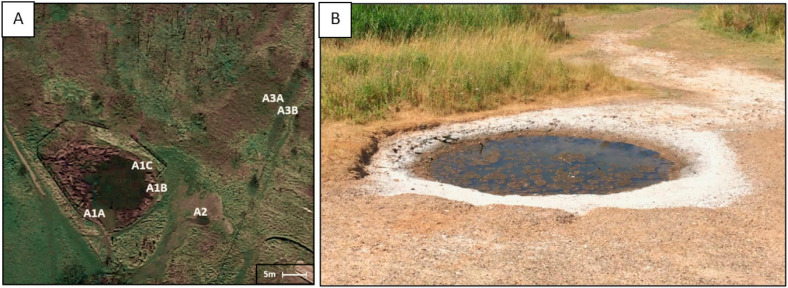
Aerial image of the Anderton Brine Springs depicting spring locations ((**A**) Source: Google Images 2017). A1A, A1B, A1C, A3A and A3B designate sampling points.; brine pool A2 ((**B**) approximately 1.5 m in diameter).

Samples from the Cheshire Salt District were collected in October 2017 from the Anderton Brine Springs (Figure 1; 53.2721°, −2.5244°), which consists of two subaerial springs (A1 and A2), and an underground spring (A3). Three samples were taken from different areas of A1 (A1A (53.2720°, −2.5247°), A1B (53.2721°, −2.5244°), and A1C (53.2721°, −2.5245°)). One sample was also taken from A2 (53.2720°, −2.5242°); and two samples from A3 (A3A (53.2722°, −2.5239°) and A3B (53.2722°, −2.5239°)). A1 and A2 brine pool samples were collected by submerging sterile 50 mL Falcon tubes below the brine pool edge. A3 underground spring samples were collected by boring approximately 10–15 cm into the ground, allowing the borehole to fill with water from the spring, and collecting the water into a 50 mL falcon tube by using a sterile syringe. Relevant physicochemical conditions were measured for all samples from the Anderton Brine Springs. Salinity was measured using a salinity refractometer (ATAGO, Saitama, Japan). The pH and temperature were measured on site using a pH meter/thermometer (HANNA). Selected ion concentrations were measured using MQuant^TM^ test strips (Merck, Feltham, UK) to measure concentrations of sulfate (SO_4_^2−^), sulfite (SO_3_^2−^), nitrate (NO_3_^−^) and nitrite (NO_2_^−^) and MColortest^TM^ chemical indicator kits (Sigma-Aldrich, Gillingham, UK) were used to measure ammonium (NH^4+^), phosphate (PO_4_^3−^), and iron (Fe—bivalent and trivalent iron in its dissolved form and colloidal iron (III) hydroxide).

### 2.2. Isolation and Identification of Microbial Strains from the Anderton Brine Springs 

Brine samples were diluted 10^0^–10^7^ in 10-fold increments in saline solution (salt/water; *w*/*v*) and spread onto nutrient agar (NA; OXOID—1 g/L Lab-Lemco powder, 2 g/L Yeast extract, 5 g/L Peptone, 5 g/L Sodium chloride, 15.0 g/L Agar) plates to produce individual microbial colonies and allow the subsequent isolation and identification of microbial strains from the Anderton Brine Springs; both saline solution and NA plates were adjusted to match the salinity of the isolation site (NaCl (*w*/*v*) A1B 4.2% A1C 1.4% A2 5% A3B 1.3%). Incubations were performed at 30 °C. Individual colonies were selected for further characterisation based on morphological differences, and then picked and purified via subculturing in the same medium. After the strains were identified as pure, DNA from each individual strain was extracted using the Griffiths technique (a phenol/chloroform-based DNA extraction technique; [27]). The 16S rRNA gene was amplified from genomic DNA using Polymerase Chain Reaction (PCR) with the forward primer 27F (Sigma-Aldrich (Gillingham, UK); 5′-AGAGTTTGATCCTGGCTCAG-3′) and the reverse primer 1492R (Sigma; 5′-TACCTTGTTACGACTT-3′) [28]. The resulting 16S rRNA gene amplicons were then sequenced as described in Kelbrick et al., 2021 [18]. PCR products were purified using the Sigma-Aldrich GenElute PCR Clean-Up Kit (Gillingham, UK) according to the manufacturer’s instructions and were sequenced by Sanger sequencing at MWG Eurofins (Konstanz, Germany). Sequence quality was assessed by analysing chromatograms using MEGA7 (7.0.26) [29], and sequences were then assigned to a genus based on the greatest level of sequence similarity of the 16S rRNA gene to the 16S rRNA gene sequence of other identified microbes using the NCBI BLAST database (www.ncbi.nlm.nih.gov) (accessed on 12 September 2021) [30] and EzTaxon-e (www.ezbiocloud.net) (accessed on 12 September 2021) [31].

### 2.3. Preparation of Simulated Martian Fluids 

The martian simulated fluids used in this study were made to contain the same elemental composition as a mineralogical simulant based on the chemistry of the Rocknest regolith at Gale Crater [32,33]—a sand shadow found on top of the sediments of an ancient lake deposit from the Noachian–Hesperian transition [34]. The composition of regolith across Gale Crater has been shown to possess a basaltic lithology similar to other sites characterised across the planet by the Viking, Pathfinder, and MER missions [9,32,35,36]. The millimolar concentration of individual components are as follows: (NH_4_)_2_SO_4_ 0.5, Fe_2_(SO_4_)_3_ 0.1102, MnCl_2_ 0.0294, MnSO_4_.H_2_O 0.0190, NaHS 0.0865, K_2_HPO_4_ 0.0397, NaOH 0.3695, KOH 0.1741, Ca(OH)_2_ 1.3818, FeO_2_ 0.6184, 3Al_2_O_3_.2SiO_2_ 0.9891, 3MgO.4SiO_2_.H_2_O 1.5197, SiO_2_ 4.6659, and TiOH 0.0923. This fluid did not contain perchlorates, as the era for which the fluids are simulating are not believed to have high concentrations of perchlorates [37,38]. 

The fluids used in this study were made in 1 L volumes in an anaerobic chamber (Coy, UK) with a headspace of CO_2_/H_2_/N_2_ (90:5:5) to prevent the oxidation of the individual components prior to the experiments. The water used for these fluids was boiled to reduce the concentration of dissolved oxygen [39]. The components comprising the fluids were prepared separately under anoxic conditions as above, and aliquots of these added individually to produce complete fluids of the desired elemental composition. 

### 2.4. Growth of Microbial Isolates from the Anderton Brine Springs in Rocknest Fluid Chemistry

For testing microbial growth, the prepared Rocknest fluid was diluted with sterilised deionised water to final concentrations of 10%, 50%, 90%, and 100%, then supplemented with yeast extract (final concentration of 4 g/L). A test group of sterile deionised water was also supplemented with 4 g/L of yeast extract as a control—designated as 0%. All isolated strains were plated on media containing 4 g/L yeast extract and 1% agar (*w*/*v*) and incubated for 24 h, to ensure they could grow with the sole substrate and low Na/Cl. A colony of each strain that grew was picked from a 24-h grown culture and used to inoculate two tubes of each dilution under oxic conditions. After inoculation, the tubes were incubated at 30 °C in a shaker at 250 rpm and the optical density (OD) of the fluids was measured every 24 h for a period of five days. The conditions of the experiment, with regard to temperature, pressure, atmosphere and nutrient supply, were selected in order to ensure that there were no confounding variables for assessing the habitability of our simulated martian fluid chemistry. 

To exclusively challenge the isolates from this proposed analogue site against the defined chemical environment, a simulation of an appropriate water chemistry that may have existed on Mars during the Noachian–Hesperian transition was used, and to remove other confounding variables, this experiment was performed under oxic conditions and standard atmospheric pressure as performed in Fox-Powell et al., 2016 [40] and Stevens et al., 2019 [23]. However, whilst an estimate of ~1 bar atmosphere is feasible for the Noachian, it is possible it was much lower during the Noachian–Hesperian transition [11], and this is therefore a caveat of this study.

In line with seeking to assess the viability of the microbes under the chemical environment, yeast extract was also supplied as the sole carbon source, as performed in Fox-Powell et al., 2016 [40]. The cultures were incubated at 30 °C, close to the ranges used in previously published simulation studies [23,40,41,42]. The temperature of 30 °C is also within the range of some models, which estimate the conditions of the Noachian as warm and wet [43,44], with some studies suggesting that these conditions may have persisted into the early Hesperian [45]; however, other models have predicted colder climates [2]. A second range of microbial habitats would be in the subsurface, where higher temperatures will have persisted beyond the climate transition due to geothermal gradient globally [46] and locally beyond the temperature increase with depth volcanic and impact activity enhancing the subsurface temperature [3]. The assumed location of a microbial habitat is therefore another important limitation to these experiments and simulation studies more broadly.

### 2.5. Analysis of Fluid Chemistry by ICP-OES 

To assess the impact of microbes on the fluid chemistry, inductively coupled plasma-optical emission spectroscopy (ICP–OES) was employed using an Agilent 5110 (Agilent, Milton Keynes, UK) at the Open University, as previously described [47]. Fluid samples were taken at the end of the growth experiments from both the biotic and abiotic test groups and filtered using 0.22 μm filters (Starlab, Milton Keynes, UK) and acidified through the addition of 1% nitric acid (Sigma-Aldrich, Gillingham, UK) prior to analysis. 

## 3. Result

### 3.1. Chemical Analysis of Environmental and Simulated Martian Fluid Samples

Salinity varied across the sample sites from Anderton over a range of 1.4–5% (*w*/*v*) NaCl. Postliminary analysis in November 2017 measured the salinity of A2 at 13%—compared to 5% at the time of sampling—indicating the fluctuating nature of the brine springs. pH and temperature also varied from 7.4 to 8.4 and 12.7 to 14.3 °C, respectively. There was also a variation in the ionic compositions of the sampled sites, with SO_3_^2-^ absent in all sites whilst SO_4_^2−^ was present in high concentrations (>16,000 mg/L) at A1 and lower concentrations (>400 mg/L) at the higher salinity sites (A2 and A3). Fe^2+^ was detected in all samples at low concentration (0.05–0.6 mg/L) except in A1B which exhibited a higher concentration (>1 mg/L).

Analysis of the Rocknest fluid chemistry following growth of the Anderton Brine Springs isolates revealed minimal changes in the abundance of specific elements relative to the uninoculated fluids (Supplementary Table S2). Of the seven isolates grown in the Rocknest fluid, aluminium was present in the fluid chemistry when five of the strains were grown in them (Strains MKS3, MKS8, MKS19, MKS13 and MKS29 had values of 20–170 μg/kg relative to being absent in the undiluted Rocknest fluid owing to the aluminium being supplied as aluminium silicate). However, this increase was not observed in strains MKS9 and MKS28.

### 3.2. The Impact of the Simulated Martian Fluid Chemistries on the Microbes of Anderton Brine Springs 

DNA was extracted from 14 isolates, followed by 16S rRNA gene amplification and sequencing. The 16S rRNA gene sequence identified the organisms (Table 1) as either *Gammaproteobacteria* or *Bacilli*. Due to the high number of species with which there was 100% sequence similarity, the isolates were identified to the genus level. 

**Figure 2:**
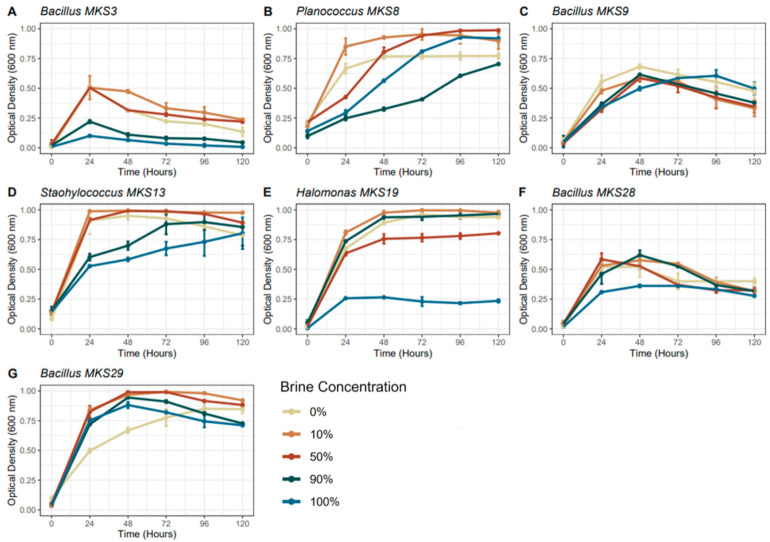
Growth curve of selected strains from the Anderton Brine Springs grown in Rocknest fluids at concentrations of 0, 10, 50, 90, and 100%. (**A**–**G**) subfigures represent the individual strains. All strains were grown in duplicate with the average optical density used to calculate the growth curves.

As the Rocknext fluid has limited Na/Cl availability, all of the isolated strains were plated on media containing 4 g/L yeast extract and 1% agar (*w*/*v*) and incubated for 24 h, to ensure they could grow with the sole substrate and low Na/Cl concentrations. Of these strains, seven of them were able to grow simultaneously in the absence of NaCl and with yeast extract as the sole supplied carbon source; these strains were therefore selected to grow in the Rocknest fluids (Table 1). All seven isolates were able to grow in all dilutions of the Rocknest fluids. However, the seven strains all exhibited a lower rate of growth in the higher concentrations of the Rocknest fluids (100% and 90%) (as shown in Figure 2). Additionally, all strains, except for strain MKS9, produced less overall biomass in both the 90% or 100% concentrations of the Rocknest fluids. *Halomonas*, *Planococcus*, and *Staphylococcus* isolates grew rapidly before plateauing and maintaining high levels of biomass, though some treatments did start to decline in growth. However, all *Bacillus* spp. grew before declining in biomass. The raw data is available in Supplementary Table S1.

## 4. Discussion

A range of techniques was applied to assess the viability of microbes from a martian analogue environment under simulated martian chemical conditions relevant to the waters modelled to have existed on the surface of Mars during the Noachian–Hesperian transition. The impact of these simulated environments on the growth of microbes was observed, with the subsequent effect that the microbes had on the fluid chemistries also examined. 

### 4.1. Variation in Viability of the Anderton Brine Springs Isolates

All seven of the tested microbial strains from the Anderton Brine Springs grew in the Rocknest fluid chemistry, with growth observed in every test group of the dilution series. This growth confirms that the Anderton Brine Springs contains microbes that are viable under a chemically accurate proxy for martian chemical conditions. This is in line with prior Mars simulation experiments, which identified that microbial growth was viable in water chemistries analogous to those proposed to have existed on early Mars [23,41,47]. Variation in growth was observed when comparing the seven isolates, with some performing better under the varied concentrations of the Rocknest fluid chemistries, achieving higher optical densities and faster growth rates. 

Intrageneric variation in growth was observed between the isolates, with *Bacillus* MKS28 reaching a higher optical density under all growth conditions than the other tested strains of the genus *Bacillus*, and *Bacillus* MKS9 not exhibiting any consistent pattern across the concentration gradient. The phenotype of these strains is clearly impacted by exposure to the simulated martian fluid chemistry. This result also shows that genetic variation at the genus and strain levels also plays a role in the relative viabilities of the tested strains under environmental stresses, and supports the importance of cultivation-dependant characterisation of analogue environments in addition to cultivation-independent techniques [48,49]. *Bacillus* has been identified in other martian analogue environments [50], and has been previously studied from an astrobiology perspective [51,52,53]. The interest in *Bacillus* for astrobiological studies is largely due to their ability to form spores that can tolerate extremes, such as those associated with fluctuation in the concentration of brines. They can also withstand the stresses of atmospheric entry, for this reason they are also relevant from a planetary protection perspective [54,55]. Although sporulation was not investigated during this study, the tendency to sporulate when exposed to environmental stressors could explain why *Bacillus* spp. rapidly decreased in OD. A phenomenon that has been observed in OD measurements of *Bacillus* sp. when they undergo the stressors of nutrient limitation [56,57,58,59,60]. For the strains that exhibited reduced growth in the higher fluid concentrations (*Bacillus* MKS3, *Staphylococcus* MKS13, *Halomonas* MKS19, *Bacillus* MKS28), this is possibly due to the changes in concentrations of salts or toxicity from some of the elements in the Rocknest fluid chemistry (e.g., Mn, Al, Fe; [61]) reaching concentrations that are inhibitory for the affected stains. Toxicity and inhibitory concentrations of compounds are aspects to consider in the context of the drying waters of Mars, with prospective organisms being subjected to multiple selective stresses due to the increase in concentration of several elements. This would lead to increased salinity, with associated reduced water activity, and increased toxicity. Strains of *Halomonas* have previously been detected in numerous environments considered appropriate physicochemical analogues for Mars (including the Great Salt Plains of Oklahoma, Colour Peak Springs, and the Antarctic dry valleys) [47,62,63], indicating that other members of the genus are also capable of survival under proxy conditions for martian chemistries. Strains of *Staphylococcus* have also been shown to be tolerant to simulated martian conditions, including simulated temperatures and air chemistry. In addition, they are also tolerant to microgravity, with members of the genus identified as highly prevalent on the International Space Station [64,65]. *Bacillus* and *Staphylococcus* have both also been studied as model organisms in space microbiology and studies pertaining to the transport of life in rocky ejecta [66,67], further enhancing the importance of considering their relative viabilities under a range of extraterrestrial conditions. 

### 4.2. Microbial Influence on Environmental Chemistry

Given the absence of liquid water on the surface of Mars today [3], there is limited potential for shifts in fluid chemistry to represent reliable biosignatures. Furthermore, given the requirement for the digestion of sediment and regolith materials with hydrofluouric acid to allow their analysis with ICP-OES, and the need to prepare samples and subsequently optimize their analysis dependent on their chemical composition, ICP-OES has limited versatility in terms of future mission payload instrumentation, but may have potential application following future Mars sample return. However, shifts in fluid chemistry, as a result of the former presence of metabolic processes within ancient martian aqueous systems, would have potentially impacted on mineral formation and alteration within these systems [68], which could act as putative biosignatures [69,70,71,72]. Therefore, using ICP-OES as an analytical technique to study the impact of microbially influenced enrichment or depletion of specific chemical species within terrestrial aqueous environment simulating martian chemical conditions has the potential to inform the identification of putative biosignatures. 

Furthermore, understanding the types of organisms that can inhabit specific aqueous environments is useful to establish chemical changes that might occur over geological timescales. In the Anderton Brine Springs isolate growth experiments, Al was the only element to shift in concentration, and this was not detected for two of the isolates. As Al can influence the formation, precipitation and adsorption of specific minerals and chemicals [73], the detection of this specific element shifting in abundance suggests that this specific chemical shift could impact on this occurring in former concentrated aqueous systems. However, the detection or altered presence of Al-minerals could only act as an ambiguous biosignature given the potential for similar shifts to occur in abiotic systems over geological timescales [73,74]. This limited impact on the fluid chemistry by the Anderton Brine Spring isolates may potentially be due to the short time scale of the experiment prohibiting the detection of a shift in fluid chemistry via ICP-OES. However, as all strains reached the stationary phase of growth, it is possible that the absence of an impact on the fluid chemistry would also occur under longer timescales. It is also possible that over longer timescales, microbes would impact the fluid chemistry through cell death, with necromass acting as a sink for specific elements [75].

Therefore, with these strains of bacteria in combination with this specific set of simulated martian chemical environments, growing cells and active metabolic processes did not have a measurable impact on the chemistry of their environment. The strain-dependent impact on the chemical environment requires further investigation, as this has potential broader implications for the ability to infer the potential former presence of life and identify putative biosignatures in an environment. It is also possible that changes did occur in all test groups, but that ICP-OES was not sufficiently sensitive to detect these shifts in chemistry. This would be similar to observations from simulation experiments performed by Stevens et al., 2019 [41], in which bulk analytical techniques were not able to identify differences between biotic and abiotic test groups, but more sensitive techniques were able to identify putative biosignatures resulting from the presence of cells. 

All isolates tested in this study grew in the fluid chemistry at variable concentrations. However, on Mars, as evaporation concentrated the brines, there would have also been periods of complete desiccation that would have further impacted survival [76], similar to other terrestrial environments, such as brine lakes [76,77]. Fluctuating environments are a greater challenge for survival, as for life to survive in such an environment requires the ability to withstand a changing gradient of selection pressures and it is therefore potentially harsher than thriving under static stressors [59,78,79]. Previous studies have identified a range of microbes capable of surviving extended periods of desiccation [25,26,80], indicating the relevance of these organisms to understanding survival under conditions analogous to former martian environments. 

Therefore, future experiments involving the isolates from the Anderton brine Springs should also consider a greater range of fluid chemistries and water activities, 

This could be tested by repeating this study in open vessels, as opposed to close, to allow for the gradual evaporation of the fluids, allowing the fluid to increase in concentration and eventually become desiccated over a generational timescale. Viability of the cells could be assessed over varying lengths of desiccation or in cycles of fluid replenishment and desiccation to represent the fluctuating conditions of the Noachian–Hesperian transition more closely [81]. This would allow for the microbial cells to be challenged using a relevant physical stressor in addition to the fluid chemistry. This approach could further assess the possibility of the formation and preservation of biosignatures by these microbes under fluctuating conditions.

## 5. Conclusions

Identifying martian analogues and developing an understanding of the interaction between their biota and martian chemical systems through simulation studies, is key to developing hypotheses with regard to habitability, the metabolisms that could have been viable within these environments and the resulting biosignature formation. This study shows that microbes from fluctuating brines are capable of growth in chemistry simulated waters that may have potentially existed on Mars, with this work supporting that the investigation of fluctuating environments and their associated microbiomes on Earth combined with simulation studies furthers our understanding of potential habitable martian conditions both past and present.

## Figures and Tables

**Table 1:** 16S rRNA gene sequencing results of isolates from the Anderton Brine Springs, including data on isolation site, closest relative of isolate, and the strain’s ability to grow in the absence of NaCl with Yeast Extract as the sole carbon source.

Strain	Isolation Site	Isolate Accession	Genus with Highest Sequence Similarity	Closed Relative Accession	Similarity to Closest Relative	Growth at 0% NaCl on Yeast Extract Agar (+ Represents Growth and—Represents no Growth)
MKS3	A1C	MW132413	*Bacillus* sp.	MK712419.1	100% MK712419.1	+
MKS8	A1B	MW130959	*Planococcus* sp.	MK696244.1	100% MK696244.1	+
MKS9	A3B	MW132410	*Bacillus* sp.	MK618601.1	100% MK618601.1	+
MKS13	A3B	MW131453	*Staphylococcus* sp.	MK120203.1	100% MK120203.1	+
MKS15	A1B	MW130887	*Halomonas* sp.	HF678757.1	99% HF678757.1	-
MKS16	A2	MW130884	*Salinivibrio* sp.	NR_042255.1	100% NR_042255.1	-
MKS19	A2	MW131523	*Halomonas* sp.	CP024811.1	100% CP024811.1	+
MKS20	A1B	MW130885	*Motilimonas* sp.	NR_156090.1	97.5% NR_156090.1	-
MKS21	A1B	MW131554	*Planococcus* sp.	MK696244.1	100% MK696244.1	-
MKS22	A1B	MW134719	*Photobacterium* sp.	JN791338.1	99% JN791338.1	-
MKS23	A1B	MW132418	*Marinobacter* sp.	MH266164.1	99% MH266164.1	-
MKS24	A1B	MW132419	*Pseudoalteromonas* sp.	LT601323.2	99% LT601323.2	-
MKS28	A2	MW131455	*Bacillus* sp.	MK618601.1	100% MK618601.1	+
MKS29	A1C	MW130923	*Bacillus* sp.	MG575987.1	99% MG575987.1	+

## Data Availability

Sequence data generated in this study was deposited to NCBI GenBank. Accession numbers for the 16S rRNA gene sequences are MW132413, MW130959, MW132410, MW131453, MW130887, MW130884, MW131523, MW130885, MW131554, MW134719, MW132418, MW132419, MW131455 and MW130923.

## Supplementary Materials

The following are available online at https://www.mdpi.com/article/10.3390/life12010012/s1, Table S1: Optical Densities of Anderton Brine Spring Isolates grown in the Rocknest Fluids, Table S2: ICP-OES analysis of Rocknest brine fluid in varying concentrations under either abiotic conditions or combined with isolates from the Anderton Brine Springs.
